# Identifying Suitable Reference Gene Candidates for Quantification of DNA Damage-Induced Cellular Responses in Human U2OS Cell Culture System

**DOI:** 10.3390/biom13101523

**Published:** 2023-10-13

**Authors:** Nikolett Barta, Nóra Ördög, Vasiliki Pantazi, Ivett Berzsenyi, Barbara N. Borsos, Hajnalka Majoros, Zoltán G. Páhi, Zsuzsanna Ujfaludi, Tibor Pankotai

**Affiliations:** 1 Department of Pathology, Albert Szent-Györgyi Medical School, University of Szeged, Állomás utca 1, H-6725 Szeged, Hungary; barta.nikolett@szte.hu (N.B.); ordog.nora@szte.hu (N.Ö.); pantazi.vasiliki@med.u-szeged.hu (V.P.); berzsenyi.ivett@szte.hu (I.B.); borsos.barbara.nikolett@szte.hu (B.N.B.); hajnalka.majoros@szte.hu (H.M.); pahi.zoltan@szte.hu (Z.G.P.); 2 Competence Centre of the Life Sciences Cluster of the Centre of Excellence for Interdisciplinary Research, Development and Innovation, University of Szeged, Dugonics tér 13, H-6720 Szeged, Hungary; 3 Genome Integrity and DNA Repair Core Group, Hungarian Centre of Excellence for Molecular Medicine (HCEMM), University of Szeged, Budapesti út 9, H-6728 Szeged, Hungary

**Keywords:** DNA damage, DNA repair, reference gene, housekeeping gene, UVB, NCS, ActD

## Abstract

DNA repair pathways trigger robust downstream responses, making it challenging to select suitable reference genes for comparative studies. In this study, our goal was to identify the most suitable housekeeping genes to perform comparable molecular analyses for DNA damage-related studies. Choosing the most applicable reference genes is important in any kind of target gene expression-related quantitative study, since using the housekeeping genes improperly may result in false data interpretation and inaccurate conclusions. We evaluated the expressional changes of eight well-known housekeeping genes (i.e., *18S rRNA*, *B2M*, *eEF1α1*, *GAPDH, GUSB*, *HPRT1*, *PPIA*, and *TBP*) following treatment with the DNA-damaging agents that are most frequently used: ultraviolet B (UVB) non-ionizing irradiation, neocarzinostatin (NCS), and actinomycin D (ActD). To reveal the significant changes in the expression of each gene and to determine which appear to be the most acceptable ones for normalization of real-time quantitative polymerase chain reaction (RT-qPCR) data, comparative and statistical algorithms (such as absolute quantification, Wilcoxon Rank Sum Test, and independent samples T-test) were conducted. Our findings clearly demonstrate that the genes commonly employed as reference candidates exhibit substantial expression variability, and therefore, careful consideration must be taken when designing the experimental setup for an accurate and reproducible normalization of RT-qPCR data. We used the U2OS cell line since it is generally accepted and used in the field of DNA repair to study DNA damage-induced cellular responses. Based on our current data in U2OS cells, we suggest using *18S rRNA*, *eEF1α1*, *GAPDH*, *GUSB*, and *HPRT1* genes for UVB-induced DNA damage-related studies. *B2M*, *HPRT1*, and *TBP* genes are recommended for NCS treatment, while *18S rRNA*, *B2M*, and *PPIA* genes can be used as suitable internal controls in RT-qPCR experiments for ActD treatment. In summary, this is the first systematic study using a U2OS cell culture system that offers convincing evidence for housekeeping gene selection following treatment with various DNA-damaging agents. Here, we unravel an indispensable issue for performing and assessing trustworthy DNA damage-related differential gene expressional analyses, and we create a “zero set” of potential reference gene candidates.

## 1. Introduction

Of various cellular processes, DNA repair pathways play an essential role in maintaining genome integrity. Any malfunction in DNA repair results in transcriptional dysregulation of several proto-oncogenes and tumor suppressors implicated in cancer development [1,2]. Moreover, chemoresistance in cancer cells may be brought on by transcriptional dysregulation of DNA repair genes [3]. By inducing apoptosis, the inappropriate expression level of DNA repair genes might also influence the overall survival rate of the damaged cells [4]. Transcriptional silencing in the close vicinity of the damaged genomic locus has been reported as an integral part of DNA damage response, which, if dysregulated, can have a significant impact on the expression of many genes, including housekeeping genes [5,6]. Based on this proof, careful consideration should be taken when deciding which reference genes are the ideal ones to use for studying DNA repair and related cellular responses. Nowadays, real-time quantitative polymerase chain reaction (RT-qPCR), which enables quick and accurate expression analysis of the desired transcripts, is the most frequently used method for studying transcriptional responses. To accurately interpret these data, it is essential to choose the appropriate reference gene(s) from which stable mRNAs are produced [7]. Since housekeeping genes are indispensable for maintaining cellular homeostasis, and since the transcribed mRNAs have a longer half-life than other transcripts, including them in expressional analyses has long been the solution for this issue [8,9,10]. Because of their role in maintaining essential cellular functions, and the assumption according to which their expression is stable, these genes are widely utilized as endogenous controls in various experimental setups and in various cell lines without confirming their expression stability. *18S rRNA* and *GAPDH* are the most frequently employed housekeeping genes to assess the relative expression of the target genes [11,12]. Several studies have already proven that housekeeping genes are not constitutively expressed under various conditions [13,14,15,16]. Since then, it has become clear that diverse cell and tissue types, as well as various experimental settings, do not all express a single gene product in exactly the same way [10]. For these reasons, in order to effectively normalize gene expression levels, reference genes that are stably expressed in the relevant experimental setup must be carefully selected in each biological model. To identify the appropriate reference genes for normalization, we tested eight well-known housekeeping genes—represented in Table 1—in U2OS cells that are commonly used as reference genes and have different functions under various stress conditions. The U2OS cell line, which is derived from a moderately differentiated sarcoma of the tibia of a 15-year-old, white female patient, is generally accepted and used in the field of DNA repair to study DNA damage-induced cellular responses in high-quality studies [5,17,18,19,20,21,22,23,24,25]. We examined the impact of DNA-damaging chemical treatments in these cells at various time points.

Finding the right housekeeping gene panel is time-consuming, expensive, and laborious, yet definitely essential. The inclusion criteria of the eight housekeeping genes were based on their widespread use in molecular biology to normalize RT-qPCR data. The inclusion process of the candidate genes should not be underestimated, since an inappropriate choice of a reference gene may result in inaccurate data interpretation and erroneous outcomes, particularly when DNA damage is induced at random genomic locations [26]. Unfortunately, researchers inadequately emphasize the importance of selecting the appropriate reference genes; therefore, the validation procedure and the comparison of molecular biology data remain controversial [27,28,29].

To follow the transcriptional changes induced by various types of DNA damage, we applied ultraviolet (UV) B irradiation, the radiomimetic drug, neocarzinostatin (NCS), and the transcription-blocking agent, actinomycin D (ActD). We chose to employ DNA-damaging substances that are commonly used, thoroughly investigated, and easily accessible to most laboratories. According to the literature data, double-strand break repair (DSBR) and nucleotide excision repair (NER) subpathways (including transcription coupled-NER [TC-NER], global genomic-NER, homologous recombination, non-homologous end joining [NHEJ], and alternative-NHEJ) can both be activated in our experimental design. These DNA-damaging factors are applied in the majority of research studies, revealing the cellular mechanism brought on by DNA-damaging agents. Based on the wavelength range, UV irradiation can be categorized into three types: UVC (190–290 nm), UVB (290–320 nm), and UVA (320–400 nm). DNA can absorb UVB light, resulting in the formation of covalent bonds between adjacent pyrimidine bases, which can be either cyclobutene–pyrimidine dimers or pyrimidine–6,4–pyrimidinone photoproducts. The formation of pyrimidine dimers may change the structure of DNA, thereby hindering DNA replication and transcription. As a result, there may be a reduction in cell viability and the development of numerous diseases [30,31]. NCS is a cytotoxic, enediyne antibiotic that can cause double-strand breaks (DSBs) at random sites by eliminating hydrogen atoms from one of the carbon atoms in the deoxyribose backbone, resulting in fiber breakage and base release [32]. ActD, on the other hand, is an antibiotic that contains a peptide that exhibits strong antibacterial and antitumor activities [33]. When applied in low doses (e.g., 5 nM), it also induces ribosomal stress; however, when used in high concentrations (e.g., 200 nM), it binds to DNA and prevents RNA polymerase from accessing the DNA template, which slows transcription [34]. Additionally, DNA–protein crosslinks produced by ActD–DNA interactions might result in DNA single-strand breaks [35]. ActD may also result in the formation of DSBR since it is a counteractor to topoisomerases I and II [36].

In this study, following UVB, NCS, and ActD treatment, we examined the expressional changes of eight well-known and frequently used housekeeping genes (i.e., *18S rRNA*, *B2M*, *eEF1α1*, *GAPDH, GUSB*, *HPRT1*, *PPIA*, and *TBP*) in the U2OS cell line. To identify the best reference genes for normalizing DNA damage-related RT-qPCR data, we assessed the expression stability of each gene. Here, we describe the first systematic report that makes use of several DNA-damaging agents to clearly demonstrate the most suitable choice of reference genes to allow for performing accurate comparative statistical analyses on the investigation of DNA damage-related gene expression utilizing human U2OS cell-based systems.

## 2. Materials and Methods

### 2.1. Cell Culturing and Treatments

The U2OS cell line (HTB-96) was procured from ATCC. The cells were cultured in DMEM (Lonza, Basel, Switzerland) supplemented with 10% fetal bovine serum (Lonza, Basel, Switzerland), 1% antibiotic-antimycotic solution (Sigma-Aldrich, St. Louis, MO, USA), and 4 mM L-Glutamine (Sigma-Aldrich, St. Louis, MO, USA), and maintained at 37 °C in a humidified atmosphere with 5% CO_2_.

A total of 6 × 10^5^ cells/mL were seeded in 60 mm diameter plates the day before each treatment and grown to reach approximately 70–80% confluency. Each experiment was carried out on passage 3 cells.

UVB irradiation was accomplished with a Vilber Lourmat VL-/6 lamp in a sterile chamber. An LM-filtered UV lamp (Vilber Lourmat, Marne-la-Vallée, France) was positioned at a height of 26 cm from the treatment platform. Before each treatment, the cells were exposed to a 16 mJ/cm^2^ dose of UVB, whose duration was determined by a UVX Digital UV Intensity Meter (Cole-Parmer, Vernon Hills, IL, USA). Before irradiation, the cells were washed twice with 1 × PBS, then the plate covers were removed. During irradiation, the cells were covered with 1 × PBS. After irradiation, the 1 × PBS was replaced by supplemented culture media, and the cells were then incubated for 0.5, 2, and 6 h at 37 °C in a humidified atmosphere with 5% CO_2_.

Following treatment with 25 ng/mL NCS (Sigma-Aldrich, St. Louis, MO, USA) or 200 nM ActD (Sigma-Aldrich, St. Louis, MO, USA), the cells were incubated for 2, 4, and 8 h, and 1, 6, and 24 h, respectively, at 37 °C in a humidified atmosphere with 5% CO_2_. The culture medium was not replaced either before or after the treatments.

### 2.2. RNA Isolation and Reverse Transcription

Following the treatments, the cells were washed two times with 1 × PBS and scraped in 1 × PBS. The cells were sedimented at 20,000 RCF, 4 °C for 10 min, then total RNA was isolated using a ReliaPrep RNA Cell Miniprep System Kit (Promega, Madison, WI, USA) according to the manufacturer’s instructions. RNA concentrations were determined with a NanoDrop One^C^ spectrophotometer (Thermo Fisher Scientific, Waltham, MA, USA), and reverse transcription was carried out using TaqMan Reverse Transcription Reagents (Thermo Fisher Scientific, Waltham, MA, USA), according to the manufacturer’s instructions, with the following thermal profile: 25 °C for 10 min, 37 °C for 60 min, and 95 °C for 5 min. In each RT-qPCR reaction, an equal amount of cDNAs corresponding to 4.16 ng transcribed RNA was utilized.

### 2.3. RT-qPCR

RT-qPCR reactions, in a final volume of 10 µL, using GoTaq qPCR Master Mix, (Promega, Madison, WI, USA) were performed with a QIAGEN Rotor-GeneQ 5-plex HRM qPCR System (Qiagen, Hilden, Germany). All the RT-qPCR amplifications were performed under the same thermal profile conditions: 95 °C for 7 min, 45 cycles of 95 °C for 15 s, and 60 °C for 30 s, followed by a melting curve analysis. The cycling conditions were optimized to ensure efficient amplification of the target gene while minimizing non-specific amplification. The primers listed in Table 2 were designed using Primer3 software (https://primer3.ut.ee/). Using NCBI BLAST (http://www.ncbi.nlm.nih.gov/tools/primer-blast/), the specificity of the primers was checked. The primers were also tested for the most suitable concentrations in RT-qPCRs. Absolute quantifications were calculated using a standard curve of a 5-step, 2-fold serial dilution from the mixture of non-treated samples, where the quantification cycle (Cq) value of the non-diluted sample was determined to be 100%. To reduce the experimental bias, 4 independent biological replicates were collected for each condition. The cells were independently treated and harvested. For each RT-qPCR measurement, 2 technical duplicates were applied. In Section 3 each box plot represents data from 4 independent measurements.

### 2.4. Statistics

Validating our findings, statistical analyses were conducted using 3 distinct software programs. Box plot graphs were created using the SigmaPlot program package (Systat Software Inc., San Jose, CA, USA, version 12.5), which also displays the related error bars, medians, and means. All 3 different statistical analysis methods were conducted using the computed absolute quantification values. All the datasets underwent descriptive statistics and Shapiro–Wilk normality tests. *p*-values were determined using a two-tailed Independent Samples T-test or a Wilcoxon Rank Sum test, depending on whether the datasets had normal or non-linear distribution, respectively. In addition to SigmaPlot, Independent Samples T-tests were performed in IBM SPSS Statistics (version 28.0) and R (version 4.2.1); both corroborated the statistical findings provided by SigmaPlot. In each case, the cut-off value for significance was set at 0.05.

Using a two-step process, the suitable reference genes were selected from the candidates. First, for each treatment, we excluded the genes whose expression levels significantly differed from the untreated samples across all time points of the examined conditions. Second, we subjectively ruled out genes with high standard deviation in raw expression data for a single time point.

## 3. Results

Preserving genome integrity is essential for the maintenance of life, but susceptibility to particular chemical modifications makes our genome vulnerable to exogenous and endogenous damaging factors. Furthermore, by specifically targeting certain proteins in the cells, DNA-damaging agents can affect the ongoing cellular processes. In this study, our objective was to identify the human reference genes that were most resistant to various DNA-damaging agents. Subsequently, the expressional levels of the desired housekeeping genes were temporally monitored following treatment with the aforementioned UVB light and substances.

**Figure 1:**
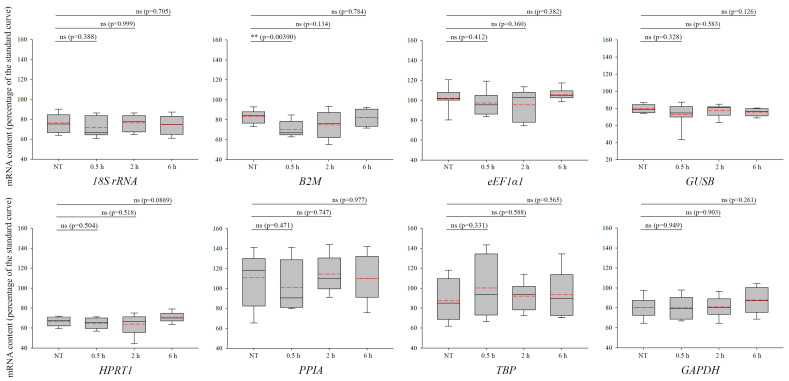
Expression changes in the proposed reference genes upon ultraviolet (UV) B irradiation. mRNA levels of *18S rRNA*, *B2M*, *eEF1α1*, *GAPDH*, *GUSB*, *HPRT1*, *PPIA*, and *TBP* are shown in the panels 0.5, 2, and 6 h following UVB irradiation, as well as in non-treated (NT) samples. In each box, the median value is depicted by the solid black line, while the mean value is represented by the red dashed line. The standard error of the mean (SEM) displays the variations of 4 independent biological replicates. The “ns” indicates non-significant (*p* > 0.05), whereas asterisk(s) indicate(s) statistical significance between the NT and the treated samples (** *p* < 0.01).

We studied the potential impacts of UVB irradiation, which plays a main role in the activation of TC-NER, on the desired housekeeping genes [29,37,38,39]. Only the *B2M* expression level was significantly altered by the UVB irradiation (Figure 1, upper row, second panel, NT versus 0.5 h, *p* = 0.0039); hence, this candidate was rejected as a reference gene. Data analyses and statistics revealed that the expression of the other genes, including *18S rRNA*, *eEF1α1*, *GAPDH*, *GUSB*, *HPRT1*, *PPIA*, and *TBP,* did not change significantly following UVB exposure (Figure 1). Although all these genes proved to be statistically appropriate (data passed the normality test), we would not recommend using *PPIA* (Figure 1, lower row, first panel, and Figure S7A) and *TBP* (Figure 1, lower row, third panel, and Figure S8A) because the distribution of the data is higher than acceptable. This might reflect the diversity of the cell populations between the independent biological duplicates. Considering the equal distribution, median, and mean values of the data, we confirmed that *18S rRNA*, *eEF1α1*, *GAPDH*, *GUSB*, and *HPRT1* genes (Figure 1, Figure S1A, and Figures S3A–S6A can be utilized as reliable reference genes in UVB-related transcriptional studies.

**Figure 2:**
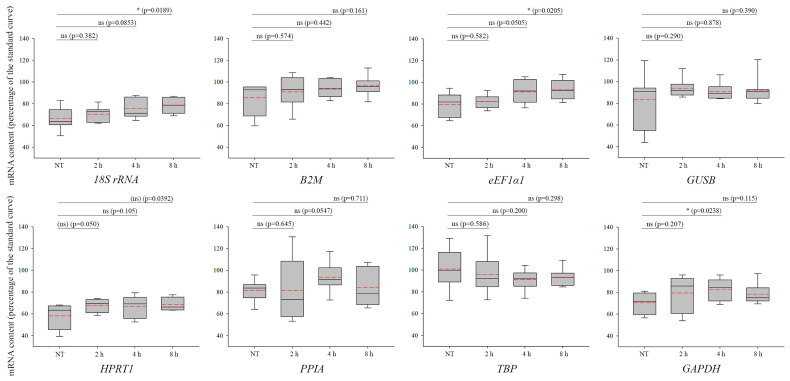
Changes in the expression level of the candidate reference genes as a result of neocarzinostatin (NCS) treatment. mRNA levels of *18S rRNA*, *B2M*, *eEF1α1*, *GAPDH*, *GUSB*, *HPRT1*, *PPIA*, and *TBP* are shown in the panels 2, 4, and 8 h following NCS treatment, as well as in the NT samples. In each box, the median value is depicted by the solid black line, while the mean value is represented by the red dashed line. SEM represents the variations in 4 independent biological replicates. The “ns” indicates non-significant (*p* > 0.05), whereas asterisk(s) indicate(s) statistical significance between the NT and the treated samples (* *p* <  0.05). Brackets indicate the cases where only 2 of the 3 statistical programs gave consistent results.

NCS is a radiomimetic drug that induces DSBs by interacting with DNA [40]. Since DSBs trigger transcriptional silencing around damaged genomic loci, careful selection of reference genes is crucial. Temporal fluctuations in the mRNA levels of *18S rRNA* (Figure 2, upper row, first panel, NT versus 8 h, *p* = 0.0189), *eEF1α1 (*Figure 2, upper row, third panel, NT versus 8 h, *p* = 0.0205), and *GAPDH* (Figure 2, lower row, fourth panel, NT versus 4 h, *p* = 0.0238) were observed upon NCS treatment. In addition, non-treated samples of *GUSB* (Figure 2, upper row, fourth panel, and Figure S5B), as well as 2 and 4 h treated samples of *PPIA* (Figure 2, lower row, first panel, and Figure S7B), showed higher distribution than acceptable. Based on the above-described criteria, *18S rRNA*, *eEF1α1*, *GAPDH*, *GUSB*, and *PPIA* are deemed as non-suitable candidate reference genes. Our experimental data demonstrate that *B2M*, *HPRT1*, and *TBP* genes (Figure 2, Figure S2B, Figure S6B, and Figure S8B) can be statistically useful normalization factors in DSB studies employing NCS treatment in the U2OS cell line.

**Figure 3:**
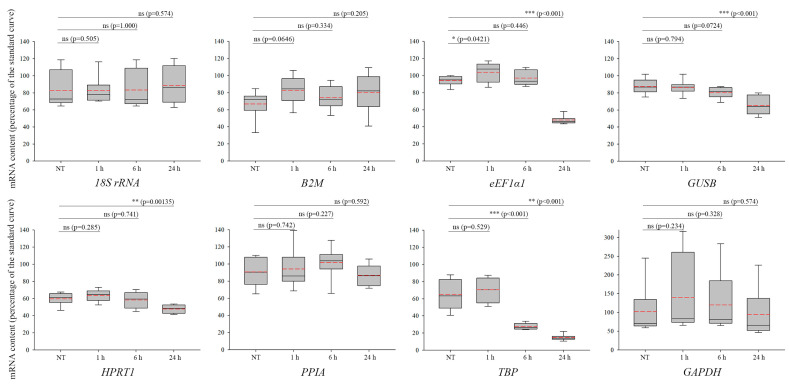
Expression profiles of housekeeping genes following treatment with actinomycin D (ActD). mRNA levels of *18S rRNA*, *B2M*, *eEF1α1*, *GAPDH*, *GUSB*, *HPRT1*, *PPIA*, and *TBP* are shown in the panels 1, 6, and 24 h following ActD treatment, as well as in the NT samples. In each box, the median value is depicted by the solid black line, while the mean value is represented by the red dashed line. SEM displays the variations of 4 independent biological replicates. The “ns” indicates non-significant (*p* > 0.05), whereas asterisk(s) indicate(s) statistical significance between the NT and the treated samples (* *p* <  0.05, ** *p*  <  0.01, and *** *p* <  0.001). In conclusion, after analyzing the expression levels of the selected housekeeping genes, we identified several acceptable control genes, which are listed in Table 3. These findings indicate that there is no common reference gene in RT-qPCR-related experiments employing various DNA-damaging agents with distinct characteristics and functions.

ActD intercalates into DNA and, by counteracting with topoisomerases, distorts DNA structure, blocking transcription and generating DSBs [41]. Being transcription factors, *eEF1α1* and *TBP* showed significant downregulation 24 h after ActD treatment (Figure 3, upper and lower row, third panel, *p* < 0.001), highlighting the importance of choosing the proper housekeeping genes for each experimental approach under various conditions [5,42]. In addition, 24 h after the treatment, a significant downregulation in the expression of *GUSB* (Figure 3, upper row, fourth panel, *p* < 0.001) and *HPRT1* (Figure 3, lower row, second panel, *p* < 0.00135) was also observed. Although based on statistical analyses, *GAPDH* seemed to be a suitable reference gene, the characteristics of the related box plots suggest avoiding using it for normalization following ActD treatments (Figure 3, lower row, fourth panel, and Figure S4C). In our experimental setup, *18S rRNA*, *B2M*, and *PPIA* (Figure 3, Figures S1C and S2C, and Figure S7C) appeared to be suitable reference genes, since their expression levels did not show any significant fluctuation following ActD treatment.

## 4. Discussion

Gene expression profile analysis has become a standard method for quantitative assessment of cellular responses to various environmental factors. For this, several approaches are currently available, including RT-qPCR, which enables precise measurements to be performed even at low expression levels. To achieve reliable results, the effect of slightly variable factors (e.g., cell number, RNA quality, RNA stability, and the efficiency of reverse transcription) should be reduced during the experiment. The most common normalization method is the use of a housekeeping gene [43,44,45]. If the expression level of a given gene remains essentially constant in the particular experimental model, examined tissues, or cells, it can be considered a suitable reference gene. Despite the rapidly expanding broad use of the RT-qPCR technique and its routine application observed nowadays, little attention is given to the proper normalization techniques required for the accurate measurement of the target gene expression. For this purpose, the widely used housekeeping genes have been utilized for a very long period without verification of their expressional stability; however, it turned out that their expressional levels rely on the current experimental setting [11,13,15,46,47,48,49,50,51,52,53,54,55,56]. When reference genes are used, the expression level of the desired gene may be over- or underestimated, depending on the applied conditions [57,58,59]. Housekeeping genes are present in all nucleated cell types, which makes them suitable candidates for the investigation of quantitative gene expression profiles. However, their careful selection and validation in the given experimental circumstances are crucial. Fortunately, the importance of validating housekeeping genes has gained momentum over time, and an increasing number of researchers have begun conducting experiments to identify the most suitable reference genes for their experimental conditions. The majority of the studies only use one housekeeping gene to normalize data [60,61,62,63,64,65]. However, by defining a set of reference genes that are consistently expressed and can be used for normalization in a given experiment, experimental bias brought on by potential variation in the individual reference genes can be minimized, which can further improve the accuracy of the analyses [10,43]. Additionally, if a single endogenous control is applied, the MIQE guidelines for RT-qPCR experiments recommend using multiple reference genes for Cq data normalization or a precise proof of invariant expression [27]. Despite all this, only a limited number of publications are currently available that employ a normalization strategy based on a number of verified reference genes [66,67,68,69]. In our experimental design, we investigated various genotoxic conditions that alter transcription, with the aim of looking for genes whose expression level remains unchanged following DNA damage. To achieve this, we overviewed the expression variations of widely used housekeeping genes in the U2OS cell line, which is frequently utilized and accepted to monitor DNA damage-induced cellular processes. Based on the data obtained with the RT-qPCR technique and the subsequent use of three distinct statistical programs, we demonstrated that for each DNA-damaging agent we used, we were able to identify more genes suitable for standards in the U2OS cell line. Regarding UVB, we identified five suitable reference genes, which are as follows: *18S rRNA*, *eEF1α1*, *GAPDH*, *GUSB*, and *HPRT1* (Table 3). Following either NCS or ActD treatment, three genes were determined as promising reference genes, respectively: *B2M*, *HPRT1*, and *TBP*; *18S rRNA*, *B2M*, and *PPIA* (Table 3). The identified reference genes particular to the treatments have proven to be promising standards on their own, although by combining them, the accuracy of the expressional data obtained from the experiments can be improved over time. In addition, we emphasize the relevance of selecting the most suitable reference genes for each experimental setup because we provided evidence showing that various reference targets were identified in the U2OS cell line after exposure to various DNA-damaging agents.

In conclusion, we shed light on the fact that careful normalization of gene expression levels is an absolute prerequisite for effective and consistent RT-qPCR expression profiling. In accordance with previous studies, our data demonstrate that there are no ideal or universal reference genes, indicating that each experimental system requires the assessment of one or more stably expressed genes that can be employed as appropriate reference genes in the current experiment. This study is the first report that provides a trustworthy set of reference gene candidates that have been pre-selected in the U2OS cell line after three different DNA-damaging treatments. For each genotoxic treatment, we identified at least three stable reference gene options that can be used to minimize the misinterpretation of RT-qPCR data. By applying the geometrical mean of the expression of the relevant reference genes for normalization, the quantitative findings of a target gene’s expression can be obtained with the greatest degree of accuracy. Future gene expression studies in the field of DNA damage may be facilitated by the work presented here. It may also increase awareness of the importance of choosing housekeeping genes carefully and encourage critical thought when designing RT-qPCR assays.

## Figures and Tables

**Table 1:** Gene symbols, names, and molecular functions of the housekeeping genes used as candidate reference genes in the present study.

Gene Symbol	Gene Name	Function
*18S*	18S ribosomal RNA	a part of the ribosomal RNA
*B2M*	Beta-2-microglobulin	a component of the major histocompatibility complex (MHC) class I molecules
*eEF1α1*	Eukaryotic Translation Elongation Factor 1 Alpha 1	a key factor in protein synthesis
*GAPDH*	Glyceraldehyde 3-phosphate dehydrogenase	a key factor in glycolysis
*GUSB*	Beta-glucuronidase	encoding a hydrolase that degrades glycosaminoglycans
*HPRT1*	Hypoxanthine-guanine phosphoribosyltransferase	generation of purine nucleotides via the purine salvage pathway
*PPIA*	Peptidyl-prolyl isomerase A	accelerating the folding of proteins and catalyzing the cis-trans isomerization of proline imidic peptide bonds in oligopeptides
*TBP*	TATA-box binding protein	a transcription factor that functions at the core of the DNA-binding multiprotein factor TFIID

**Table 2:** Primer sequences of the 8 housekeeping genes tested as prospective reference genes.

Gene	Accession Number	Primer	Sequence (5′-3′)	Primer Length (bp)	Amplicon Length (bp)
*18S rRNA*	NR_145820.1	forward	AAACGGCTACCACATCCAAG	20	250
		reverse	CGCTCCCAAGATCCAACTAC	20	
*B2M*	NM_004048.4	forward	AGGCTATCCAGCGTACTCCA	20	112
		reverse	TTCAATGTCGGATGGATGAA	20	
*eEF1α1*	NM_001402.6	forward	TCTGGTTGGAATGGTGACAA	20	141
		reverse	ACGAGTTGGTGGTAGGATGC	20	
*GAPDH*	NM_001289745.3	forward	TCGGAGTCAACGGATTTG	18	220
		reverse	TCCTGGAAGATGGTGATGG	19	
*GUSB*	NM_000181.4	forward	TGCGTAGGGACAAGAACCAC	20	129
		reverse	GGGAGGGGTCCAAGGATTTG	20	
*HPRT1*	NM_000194.3	forward	GCCCTGGCGTCGTGATTAG	19	140
		reverse	TCTCGAGCAAGACGTTCAGT	20	
*PPIA*	NM_001300981.2	forward	TTCATCTGCACTGCCAAGAC	20	158
		reverse	TCGAGTTGTCCACAGTCAGC	20	
*TBP*	NM_001172085.2	forward	ACTCCACTGTATCCCTCCCC	20	172
		reverse	TATATTCGGCGTTTCGGGCA	20	

**Table 3:** Summary of the results obtained from real-time quantitative polymerase chain reactions (RT-qPCRs).

Treatment	Recommended Genes	Not Recommended Genes
UVB	*18S rRNA, eEF1α1, GAPDH, GUSB, **HPRT1***	*B2M, PPIA, TBP*
NCS	*B2M*, ***HPRT1***, *TBP*	*18S rRNA*, *eEF1α1*, *GAPDH*, *GUSB*, *PPIA*
ActD	*18S rRNA*, ***B2M***, *PPIA*	*eEF1α1, GAPDH, GUSB, HPRT1, TBP*

Bold letters represent the most recommended genes for normalization.

## Data Availability

All relevant data are included in the paper and its Supplementary files.

## Supplementary Materials

The following supporting information can be downloaded at: https://www.mdpi.com/article/10.3390/biom13101523/s1, Figure S1: The calculated values of *18S rRNA* expression obtained from RT-qPCR measurements and the result of statistical tests; Figure S2: The calculated values of *B2M* expression obtained from RT-qPCR measurements and the result of statistical tests; Figure S3: The calculated values of *eEF1α1* expression obtained from RT-qPCR measurements and the result of statistical tests; Figure S4: The calculated values of *GAPDH* expression obtained from RT-qPCR measurements and the result of statistical tests; Figure S5: The calculated values of *GUSB* expression obtained from RT-qPCR measurements and the result of statistical tests; Figure S6: The calculated values of *HPRT1* expression obtained from RT-qPCR measurements and the result of statistical tests; Figure S7: The calculated values of *PPIA* expression obtained from RT-qPCR measurements and the result of statistical tests; and Figure S8: The calculated values of *TBP* expression obtained from RT-qPCR measurements and the result of statistical tests.
