# Supplementary material for: Identifying Suitable Reference Gene Candidates for Quantification of DNA Damage-Induced Cellular Responses in Human U2OS Cell Culture System
Source: Biomolecules. 2023 Oct 13;13(10):1523. doi: 10.3390/biom13101523 (PMC10605043; doi:10.3390/biom13101523)
Supplement: Supplementary file 1 [file biomolecules-13-01523-s001.zip › biomolecules-2587979-SI.pdf]

A

UVB	Calculated concentration	Mean	Median	Standard deviation	P-value	Normality
NT	75.503581926333	76.339	75.339	9.35		Passed
	90.2721697428242					
	74.1990180075756					
	75.1753062217693					
	64.2614657260696					
	82.0204587583786					
	85.3008460605008					
	63.9820686776209					
0.5 h	67.7109364198647	71.964	66.981	10.254	0.388	Passed
	64.2614657260696					
	66.2517070642947					
	64.8239253658498					
	80.9552737763268					
	60.7225616836061					
	84.5607137269933					
	86.4232087669872					
2 h	74.5230304544622	76.334	77.563	8.443	0.999	Passed
	71.0353727161175					
	64.5420828462086					
	66.2517070642947					
	83.4625389094283					
	80.6032951170495					
	83.8270033212439					
	86.4232087669872					
6 h	87.1796429118128	74.529	74.686	9.391	0.705	Passed
	71.6571221533289					
	74.5230304544622					
	74.8484577996664					
	80.6032951170495					
	60.9877251092234					
	83.8270033212439					
	62.6031996531911					

B

NCS	Calculated concentration	Mean	Median	Standard deviation	P-value	Normality
NT	60.7225616836061	66.445	63.861	10.274		Passed
	50.567491422219					
	74.8484577996664					
	73.8764143072884					
	62.3310124410117					
	60.7225616836061					
	65.391308031316					
	83.0996591219251					
2 h	74.8484577996664	70.42	72.601	7.025	0.382	Passed
	73.5552132286293					
	72.9169945693451					
	72.2843135604758					
	81.6638488704686					
	62.6031996531911					
	63.4269136002678					
	62.0600086488313					
4 h	72.2843135604758	75.497	71.352	9.252	0.0853	Passed
	70.4190180303426					
	69.5044988775332					
	68.3035881510008					
	87.5603391821552					
	86.8006018389216					
	64.5420828462086					
	84.5607137269933					
8 h	73.2354086732571	78.569	78.714	7.84	0.0189	Failed
	70.7265239644109					
	72.5999648714656					
	68.9014271693462					
	86.0474565308043					
	86.0474565308043					
	86.8006018389216					
	84.1930592771126					

C

ActD	Calculated concentration	Mean	Median	Standard deviation	P-value	Normality
NT	71.0353727161175	82.735	72.779	21.93		Failed
	74.5230304544622					
	68.6018564208813					
	68.6018564208813					
	64.5420828462086					
	79.5565145849448					
	116.227834662543					
	118.787815013076					
1 h	74.1990180075756	82.711	77.931	15.339	0.505	Failed
	72.9169945693451					
	71.0353727161175					
	70.4190180303426					
	84.5607137269933					
	81.6638488704686					
	116.227834662543					
	90.6663704666284					
6 h	66.2517070642947	83.300	72.301	22.214	1.000	Failed
	69.8080112872074					
	73.8764143072884					
	70.7265239644109					
	85.6733379963204					
	64.5420828462086					
	116.735378645153					
	118.787815013076					
24 h	91.8593311187661	88.433	86.584	21.864	0.574	Passed
	94.2925487995782					
	70.4190180303426					
	68.6018564208813					
	62.8765754531309					
	81.308789456844					
	117.757125321493					
	120.350789118411					

Figure S1 The calculated values of *18S rRNA* expression obtained from RT-qPCR measurements and the result of statistical test. A) UVB**; B)** NCS; **C)** ActD

A

UVB	Calculated concentration	Mean	Median	Standard deviation	P-value	Normality
NT	87.2159998779462	83.179	84.027	6.706		Passed
	75.1106689611481					
	85.2339056779069					
	88.2242654860911					
	82.8195128265649					
	72.9830337113378					
	92.9076646946749					
	80.9373343701653					
0.5 h	66.5712656724714	70.385	66.956	8.063	0.00390	Failed
	65.4333240261037					
	64.6855232948805					
	62.8531977222538					
	70.5092751655766					
	67.3408666374567					
	84.7454608952117					
	80.9373343701653					
2 h	93.4431537180767	74.747	75.98	13.403	0.134	Passed
	90.796220193801					
	59.3427873723955					
	55.0707098552879					
	70.5092751655766					
	75.5435819926106					
	76.4169079390632					
	76.8573496995804					
6 h	90.796220193801	82.097	82.008	8.649	0.784	Passed
	77.3003300206027					
	89.7585611589928					
	92.3752443657977					
	75.1106689611481					
	72.9830337113378					
	71.7354919574075					
	86.7161964280267					

B

NCS	Calculated concentration	Mean	Median	Standard deviation	P-value	Normality
NT	59.6848195541365	85.504	92.914	15.504		Failed
	61.4247790968462					
	95.6161520283721					
	93.9817291229425					
	95.6161520283721					
	91.8458751458635					
	95.0682103351199					
	90.796220193801					
2 h	65.8104600449221	91.024	93.175	14.018	0.574	Passed
	108.503068818176					
	83.7769525920115					
	96.167251876155					
	92.9076646946749					
	80.9373343701653					
	93.4431537180767					
	106.648362293977					
4 h	103.627369163579	94.302	93.722	8.289	0.442	Passed
	101.856005830111					
	82.8195128265649					
	88.2242654860911					
	92.3752443657977					
	86.2192571714764					
	95.0682103351199					
	104.224643014918					
8 h	94.5234086981534	96.465	95.901	9.017	0.161	Passed
	81.8730151027667					
	93.9817291229425					
	97.2789989504558					
	101.856005830111					
	90.2758997920855					
	98.9707642072314					
	112.957124173375					

C

ActD	Calculated concentration	Mean	Median	Standard deviation	P-value	Normality
NT	73.8267582913712	66.814	71.949	15.792		Passed
	84.7454608952117					
	70.915667286434					
	72.9830337113378					
	64.3148338829851					
	76.8573496995804					
	33.2106699060014					
	57.6618037254408					
1 h	99.5411989291117	82.77	84.531	16.033	0.0646	Passed
	106.037198981312					
	87.2159998779462					
	74.2522712867553					
	82.3449040588009					
	86.7161964280267					
	69.7034642380163					
	56.3513661120679					
6 h	94.5234086981534	74.209	72.371	13.682	0.334	Passed
	88.7327609468341					
	65.8104600449221					
	70.915667286434					
	73.8267582913712					
	82.3449040588009					
	64.3148338829851					
	53.2040891874697					
24 h	99.5411989291117	79.842	82.100	22.802	0.205	Passed
	109.128444588286					
	63.2154627471747					
	40.8443140042543					
	90.796220193801					
	95.6161520283721					
	66.189769750907					
	73.4036837575468					

Figure S2 The calculated values of *B2M* expression obtained from RT-qPCR measurements and the result of statistical test. A) UVB**; B)** NCS; **C)** ActD

A

UVB	Calculated concentration	Mean	Median	Standard deviation	P-value	Normality
NT	101.157748089812	102.057	101.426	11.217		Passed
	101.693256000083					
	120.411551140436					
	100.095177235046					
	80.1875888935223					
	100.095177235046					
	109.49496572516					
	103.316849038732					
0.5 h	91.9867243219053	97.193	95.45	11.769	0.412	Passed
	95.4500484956238					
	95.4500484956238					
	83.6471512193183					
	84.0899617009217					
	101.693256000083					
	106.080645147206					
	119.146736460048					
2 h	113.617479756308	95.54	102.796	15.913	0.360	Passed
	108.918374684741					
	74.4740815376555					
	77.6871439366018					
	100.625060117984					
	78.5118399630966					
	105.522033624305					
	104.966363701477					
6 h	107.774285451105	106.072	104.693	5.719	0.382	Passed
	103.863786776762					
	117.274381527681					
	110.074609117662					
	105.522033624305					
	98.5222117945278					
	103.316849038732					
	102.231598776959					

B

NCS	Calculated concentration	Mean	Median	Standard deviation	P-value	Normality
NT	64.579422551823	79.71	81.731	10.923		Passed
	64.9212924754845					
	89.1183534916045					
	85.8847695088408					
	75.264668963177					
	78.9274651835476					
	94.4474318742317					
	84.5351163283779					
2 h	92.4736827550899	82.204	82.334	6.100	0.582	Passed
	87.7178855167187					
	75.264668963177					
	73.6917985195805					
	81.8991048430262					
	80.6120851774837					
	82.7685133823933					
	83.2066725394979					
4 h	76.4663139629669	91.27	92.033	10.688	0.0505	Passed
	81.0388286557751					
	95.4500484956238					
	83.2066725394979					
	104.966363701477					
	94.9474167773012					
	104.966363701477					
	89.1183534916045					
8 h	86.3394254725436	93.294	92.692	9.853	0.0205	Passed
	81.467831224572					
	85.4325077242076					
	84.5351163283779					
	107.206755364519					
	100.095177235046					
	99.0437677282019					
	102.231598776959					

C

ActD	Calculated concentration	Mean	Median	Standard deviation	P-value	Normality
NT	89.1183534916045	94.097	94.959	5.537		Passed
	93.4553468356448					
	96.9739650302275					
	100.095177235046					
	99.5680846696425					
	83.6471512193183					
	96.4633084989432					
	93.4553468356448					
1 h	117.274381527681	104.005	107.78	11.236	0.0421	Passed
	108.918374684741					
	114.823598032092					
	98.0034023293917					
	109.49496572516					
	90.541180766844					
	106.642213842394					
	86.3394254725436					
6 h	107.774285451105	97.006	93.488	8.909	0.446	Passed
	90.541180766844					
	89.5901273709785					
	91.0204867955118					
	104.41361988851					
	109.49496572516					
	95.9553410408844					
	87.2559707013803					
24 h	45.8193749995694	47.887	46.432	4.676	<0.001	Passed
	49.0747572682667					
	58.1077632572565					
	44.3906149292417					
	47.0450745006164					
	43.4629461152137					
	45.3380838144898					
	49.8582648223378					

Figure S3 The calculated values of *eEF1α1* expression obtained from RT-qPCR measurements and the result of statistical test. A) UVB**; B)** NCS; **C)** ActD

A

UVB	Calculated concentration	Mean	Median	Standard deviation	P-value	Normality
NT	89.1281096149595	80.287	80.21	10.178		Passed
	79.8848214043385					
	97.6427609327752					
	82.3517287573055					
	70.8770041552326					
	64.6963783845983					
	80.5342998947769					
	77.17720431557					
0.5 h	75.170487167153	79.935	79.096	11.386	0.949	Passed
	83.8682906497686					
	66.9661371105057					
	72.4765016951278					
	83.0212653628452					
	92.627471082903					
	97.8396828974336					
	67.5105762788049					
2 h	89.6719028651134	80.925	80.312	10.404	0.903	Passed
	84.379992407545					
	72.4765016951278					
	64.5661819247402					
	87.1611367349924					
	96.656630683114					
	76.2439974637425					
	76.2439974637425					
6 h	68.6154647414833	87.152	87.628	13.069	0.261	Passed
	89.6719028651134					
	104.399554420506					
	101.890075392473					
	85.5850303715745					
	76.2439974637425					
	95.4878836310802					
	75.3220747085083					

B

NCS	Calculated concentration	Mean	Median	Standard deviation	P-value	Normality
NT	57.1704088394389	70.403	71.674	9.706		Passed
	56.4791194612542					
	66.5600374049563					
	71.1659336637744					
	81.0256604590892					
	72.1822522434096					
	79.5604999089465					
	79.0780244572101					
2 h	90.2190139361004	79.365	85.762	16.493	0.207	Passed
	86.4582107277953					
	56.4791194612542					
	53.7965461830841					
	85.0660206087192					
	73.0657443819995					
	93.7612074235655					
	96.0704798720268					
4 h	69.0341051316338	82.844	84.466	9.919	0.0238	Passed
	69.0341051316338					
	84.8948161878033					
	84.8948161878033					
	93.7612074235655					
	81.0256604590892					
	96.0704798720268					
	84.0374242246736					
8 h	72.0369857357549	78.371	75.062	9.264	0.115	Passed
	69.4552997532089					
	72.4765016951278					
	72.4765016951278					
	97.2463577516927					
	77.6480823726723					
	85.5850303715745					
	80.0459190680292					

C

ActD	Calculated concentration	Mean	Median	Standard deviation	P-value	Normality
NT	59.2954596799313	101.702	70.234	64.886		Failed
	65.7552111354986					
	148.8647234458					
	245.137950831289					
	63.1412538102463					
	70.4471876679475					
	90.9525187811112					
	70.019977699024					
1 h	83.8682906497686	139.641	82.943	108.169	0.234	Failed
	109.605454702434					
	316.491187441543					
	310.768180684426					
	65.4882453591185					
	72.1822522434096					
	76.7091817848102					
	82.0173936319233					
6 h	70.305414121776	120.274	81.471	82.376	0.328	Failed
	64.960116611233					
	215.742156849635					
	283.668533215911					
	87.1611367349924					
	91.5074435896996					
	73.0657443819995					
	75.7816341407292					
24 h	65.3564547933732	94.33	65.958	63.719	0.574	Failed
	66.5600374049563					
	156.287891524754					
	226.500181013728					
	81.0256604590892					
	64.3040428107578					
	46.5831222518803					
	48.0216474093175					

Figure S4 The calculated values of *GAPDH* expression obtained from RT-qPCR measurements and the result of statistical test. A) UVB**; B)** NCS; **C)** ActD

A

UVB	Calculated concentration	Mean	Median	Standard deviation	P-value	Normality
NT	73.9639914244113	79.473	78.817	4.811		Passed
	74.3726309487969					
	76.4499441173747					
	86.778411843397					
	81.2265546361391					
	77.2970257191267					
	85.3558479990144					
	80.3364101659495					
0.5 h	73.9639914244113	73.109	74.578	13.464	0.328	Passed
	68.4732691732755					
	43.1047772492053					
	74.3726309487969					
	80.3364101659495					
	74.783528142863					
	82.5802985510313					
	87.2578490433059					
2 h	81.675318690306	77.729	81.003	7.33	0.583	Failed
	84.8868613209195					
	69.614463354062					
	63.3901510854431					
	81.2265546361391					
	80.780256310659					
	78.585279033271					
	81.675318690306					
6 h	75.6121455020422	75.726	76.24	4.387	0.126	Passed
	76.4499441173747					
	76.0298908208207					
	80.3364101659495					
	79.0194504514088					
	79.8950027285326					
	69.614463354062					
	68.8515733130229					

B

NCS	Calculated concentration	Mean	Median	Standard deviation	P-value	Normality
NT	43.8231732240844	83.217	90.967	26.213		Failed
	43.8231732240844					
	88.7121121558819					
	88.7121121558819					
	93.7369835981792					
	94.2548658224511					
	93.221946871547					
	119.452271862274					
2 h	91.1899435360926	93.909	91.95	8.305	0.290	Passed
	87.2578490433059					
	89.2022327486254					
	85.827425755408					
	92.7097400080017					
	94.7756092652934					
	111.810026027461					
	98.5022664045449					
4 h	83.4953057806295	90.95	89.224	7.557	0.878	Passed
	87.2578490433059					
	85.3558479990144					
	84.4204514844753					
	91.1899435360926					
	106.400948510201					
	96.3551654202706					
	92.2003474588952					
8 h	84.4204514844753	92.272	90.694	12.102	0.390	Failed
	83.4953057806295					
	120.112227046149					
	89.6950611812525					
	92.2003474588952					
	88.2246845248279					
	91.6937537610101					
	92.7097400080017					

C

ActD	Calculated concentration	Mean	Median	Standard deviation	P-value	Normality
NT	89.6950611812525	87.740	86.784	8.555		Passed
	84.4204514844753					
	101.812958130553					
	96.8875126910325					
	87.7399350590183					
	85.827425755408					
	80.3364101659495					
	75.1966954798828					
1 h	87.7399350590183	86.630	86.784	8.102	0.794	Passed
	83.0365418293297					
	101.812958130553					
	81.675318690306					
	89.6950611812525					
	89.6950611812525					
	73.5575971649675					
	85.827425755408					
6 h	68.8515733130229	80.354	81.938	6.513	0.0724	Passed
	75.1966954798828					
	86.778411843397					
	84.4204514844753					
	84.4204514844753					
	87.2578490433059					
	76.4499441173747					
	79.4560205989607					
24 h	79.8950027285326	65.219	64.045	11.083	<0.001	Passed
	79.8950027285326					
	51.1331295640502					
	59.9920541157604					
	57.7223620068431					
	54.628092943594					
	70.3858063786238					
	68.0970436210646					

Figure S5 The calculated values of *GUSB* expression obtained from RT-qPCR measurements and the result of statistical test. A) UVB**; B)** NCS; **C)** ActD

A

UVB	Calculated concentration	Mean	Median	Standard deviation	P-value	Normality
NT	71.810850556618	66.553	67.632	4.56		Passed
	69.1013366301874					
	71.4664349001102					
	68.7699162106187					
	61.5709110771565					
	59.533293621218					
	66.4940560801979					
	63.6782690874					
0.5 h	63.9851515124667	64.841	65.723	5.394	0.504	Passed
	67.4600539007076					
	57.0122701839522					
	60.689243149721					
	59.247762977204					
	68.7699162106187					
	71.123671112972					
	70.4430674943457					
2 h	74.986251864426	63.919	66.494	10.275	0.518	Passed
	71.4664349001102					
	44.4012081305882					
	53.0455604252518					
	70.7825512725894					
	66.4940560801979					
	71.4664349001102					
	74.2686860587312					
6 h	68.4400853333059	70.888	70.108	4.919	0.0889	Passed
	70.7825512725894					
	66.8145085277104					
	63.6782690874					
	69.4343542524364					
	74.986251864426					
	79.058601456943					
	73.9124822529188					

B

NCS	Calculated concentration	Mean	Median	Standard deviation	P-value	Normality
NT	39.3727696751905	58.280	63.379	11.504		Failed
	42.1140788555234					
	62.4653875072842					
	64.2935128851268					
	55.9263560512708					
	66.4940560801979					
	67.4600539007076					
	68.1118363745647					
2 h	58.6808034650361	67.829	69.611	6.091	0.050	Passed
	73.9124822529188					
	64.2935128851268					
	60.1084896839194					
	68.4400853333059					
	73.2051916688447					
	73.2051916688447					
	70.7825512725894					
4 h	52.5379516388585	66.851	69.273	9.909	0.105	Passed
	63.9851515124667					
	68.4400853333059					
	53.0455604252518					
	70.1052119314408					
	70.4430674943457					
	76.8106447164485					
	79.4396057726255					
8 h	63.9851515124667	68.651	66.336	5.838	0.0392	Passed
	65.8577546284148					
	76.4422492940054					
	66.8145085277104					
	71.810850556618					
	63.3728585166712					
	77.5527703024366					
	63.3728585166712					

C

ActD	Calculated concentration	Mean	Median	Standard deviation	P-value	Normality
NT	46.1422119612959	59.735	61.276	6.941		Passed
	54.5980031309658					
	57.5631087373191					
	61.5709110771565					
	66.4940560801979					
	63.068912741045					
	67.4600539007076					
	60.9817206680356					
1 h	52.5379516388585	63.544	64.604	6.778	0.285	Passed
	64.914701017335					
	59.533293621218					
	57.2870273967873					
	69.7689767747083					
	64.2935128851268					
	73.2051916688447					
	66.8145085277104					
6 h	44.8302016994371	58.347	59.259	9.353	0.741	Passed
	47.0381487542459					
	60.3981683976417					
	53.5580736030799					
	58.1192693575128					
	67.7851617472756					
	70.4430674943457					
	64.60336033282					
24 h	47.0381487542459	47.797	48.555	4.855	0.00135	Passed
	41.5110240692482					
	42.5209747429929					
	43.346598485875					
	51.5372599631422					
	53.5580736030799					
	52.7911459269262					
	50.0718466015884					

Figure S6 The calculated values of *HPRT1* expression obtained from RT-qPCR measurements and the result of statistical test. A) UVB**; B)** NCS; **C)** ActD

A

UVB	Calculated concentration	Mean	Median	Standard deviation	P-value	Normality
NT	126.456780110011	110.666	118.018	27.513		Passed
	112.675955964875					
	131.415242905504					
	141.242382486956					
	122.271840532764					
	113.764603380294					
	72.0529286399155					
	65.4474275458265					
0.5 h	105.341600307972	100.941	90.471	24.99	0.471	Failed
	141.242382486956					
	85.2569882600592					
	83.6330962774608					
	136.568130652139					
	80.0915318347463					
	79.7074007834669					
	95.6843376647717					
2 h	143.984865818707	114.477	110.29	17.842	0.747	Passed
	133.324388560198					
	107.387003238011					
	107.904529616266					
	122.271840532764					
	112.675955964875					
	97.0743996806848					
	91.1929629586363					
6 h	141.923066894384	110.299	110.249	23.16	0.977	Passed
	134.612536060482					
	102.839545243443					
	95.2254211679069					
	124.645975538838					
	117.658369836262					
	75.6016312009135					
	89.887120487868					

B

NCS	Calculated concentration	Mean	Median	Standard deviation	P-value	Normality
NT	87.3312650254566	81.676	83.649	9.589		Passed
	80.4775141151853					
	85.2569882600592					
	82.0401346059719					
	64.2008487604115					
	72.7490863415785					
	95.6843376647717					
	85.6678642415533					
2 h	130.784955604775	81.392	73.431	28.702	0.645	Passed
	114.863769039689					
	56.9301002747723					
	64.8211415946032					
	53.2243797470625					
	59.4474902439056					
	89.0269638267365					
	82.0401346059719					
4 h	96.6088162369995	93.705	91.637	13.096	0.0547	Passed
	117.094062571041					
	90.7555877714209					
	92.517776162355					
	104.333555120771					
	90.3203103014874					
	85.2569882600592					
	72.7490863415785					
8 h	77.8142046935308	84.258	78.953	16.791	0.711	Passed
	66.3982203849786					
	80.0915318347463					
	95.6843376647717					
	107.387003238011					
	106.359384980214					
	74.8781766468359					
	65.4474275458265					

C

ActD	Calculated concentration	Mean	Median	Standard deviation	P-value	Normality
NT	109.999696581609	90.910	90.54	16.705		Passed
	108.424550091051					
	89.887120487868					
	106.871958994224					
	65.1335318246335					
	81.2550680927892					
	74.5190496323522					
	91.1929629586363					
1 h	139.219854698187	94.225	86.117	22.356	0.742	Passed
	109.472121466983					
	79.3251120825731					
	102.839545243443					
	82.0401346059719					
	68.6708003930744					
	83.6330962774608					
	88.5999770976333					
6 h	100.396918548407	101.835	103.584	17.867	0.227	Passed
	92.0740469714511					
	127.678574464006					
	111.59772614122					
	103.833155674111					
	65.7628360135422					
	103.335156218575					
	109.999696581609					
24 h	95.2254211679069	86.767	86.147	13.344	0.592	Passed
	96.1454658016432					
	105.849269348387					
	98.012308690737					
	71.7073519404547					
	74.1616450451272					
	77.0695773638921					
	75.9659754623772					

Figure S7 The calculated values of *PPIA* expression obtained from RT-qPCR measurements and the result of statistical test. A) UVB**; B)** NCS; **C)** ActD

A

UVB	Calculated concentration	Mean	Median	Standard deviation	P-value	Normality
NT	82.8023035887828	87.171	84.92	20.831		Passed
	94.2670399650554					
	114.508057333386					
	117.986517226557					
	87.0368124089072					
	70.9405725197712					
	67.826621775642					
	62.0027828507649					
0.5 h	143.320580386881	100.234	93.377	30.174	0.331	Passed
	103.121417646825					
	141.898056297716					
	111.132148846195					
	69.8870198736464					
	83.6323943917621					
	82.3903529578754					
	66.4868815427891					
2 h	102.608377173089	92.083	93.600	13.934	0.588	Passed
	99.583293179681					
	113.938367066111					
	96.1665627452279					
	91.0327116542487					
	72.3700566065712					
	84.8931597037258					
	76.0710604401763					
6 h	134.322829832965	93.739	89.724	23.661	0.565	Passed
	99.0878552670241					
	103.63702331587					
	116.815445615638					
	80.3611391331635					
	72.7319058936244					
	72.3700566065712					
	70.5876353146473					

B

NCS	Calculated concentration	Mean	Median	Standard deviation	P-value	Normality
NT	105.725355427823	100.944	99.857	17.929		Passed
	72.0100075597965					
	129.068833858837					
	119.765173801924					
	102.097889132529					
	93.7980510009292					
	97.6162816892055					
	87.4719952731238					
2 h	111.687808060991	95.911	92.189	18.225	0.586	Passed
	131.669628263924					
	96.1665627452279					
	84.0505552127457					
	86.6037946276378					
	72.7319058936244					
	93.7980510009292					
	90.5798138316783					
4 h	97.1306298765651	91.349	91.946	9.249	0.200	Passed
	90.5798138316783					
	92.4050373653845					
	97.6162816892055					
	74.1974861445957					
	104.155207006164					
	83.2163139671753					
	91.4878739596992					
8 h	87.9093540456724	93.445	93.333	7.972	0.298	Passed
	108.93701945489					
	97.1306298765651					
	84.4708068320791					
	92.8670612805042					
	93.7980510009292					
	85.3176243339179					
	97.1306298765651					

C

ActD	Calculated concentration	Mean	Median	Standard deviation	P-value	Normality
NT	82.3903529578754	65.078	63.331	17.702		Passed
	66.4868815427891					
	87.9093540456724					
	82.3903529578754					
	60.1748265852429					
	47.8383597184801					
	52.856377596673					
	40.5785419814553					
1 h	84.0505552127457	70.344	70.428	14.757	0.529	Passed
	84.0505552127457					
	87.4719952731238					
	77.6039261403358					
	63.2521665006893					
	62.9374799670196					
	50.7889167231551					
	52.5934112641593					
6 h	24.0359105138773	27.587	26.527	3.685	<0.001	Passed
	28.477802644914					
	32.0990231066431					
	33.7405667663159					
	24.6428456871515					
	25.1394099875513					
	24.6428456871515					
	27.9152969952468					
24 h	21.9721003523066	14.675	14.030	3.433	<0.001	Passed
	16.617758704166					
	13.3433842608723					
	14.3799319251978					
	13.6803204977484					
	14.4518313869226					
	10.4503120372559					
	12.5056787282804					

Figure S8 The calculated values of *TBP* expression obtained from RT-qPCR measurements and the result of statistical test. A) UVB**; B)** NCS; **C)** ActD
